# A case of bilateral inferior concha bullosa connecting to maxillary sinus^☆^

**DOI:** 10.1016/j.bjorl.2016.01.005

**Published:** 2016-04-11

**Authors:** Soo Kweon Koo, Ji Seung Moon, Sung Hoon Jung, Mi Jin Mun

**Affiliations:** Busan Saint Mary's Medical Center, Department of Otorhinolaryngology, Head and Neck Surgery, Busan, Republic of Korea

## Introduction

Nasal turbinates are important structures for the maintenance of normal nasal functions including humidification, filtration, lubrication, and thermoregulation of air inhaled through the nose arising from the lateral nasal wall. In general, individuals have three turbinates located at each side of the nasal cavity (superior, middle, and inferior). The superior and middle turbinates are part of the ethmoid bone. However, the inferior turbinate is a separate bone in itself. It articulates with the ethmoid, palatine, and lacrimal bones, creating a medial wall of inferior meatus, which is anatomically significant for the nasolacrimal duct orifice. Occasionally, individuals can have a fourth, supreme turbinate. Turbinates are composed of a pseudostratified ciliated columnar epithelium with a thick, vascular, and erectile glandular tissue layer.^1^ Concha bullosa is an air-filled cavity within the nasal turbinate. Although concha bullosa is most commonly present in the middle turbinate followed by the superior turbinate, inferior concha bullosa (ICB) is rare.^2^, ^3^ Specifically, cases of bilateral ICB that connects to the maxillary sinus are extremely rare. The cause of intranasal turbinate pneumatization is still unknown. Although this entity is considered to be a normal and asymptomatic variant in most cases, it may result in complications in a few individuals due to inferior turbinate hypertrophy or impaired ventilation and drainage of the osteomeatal complex.

## Case report

**Figure 1:**
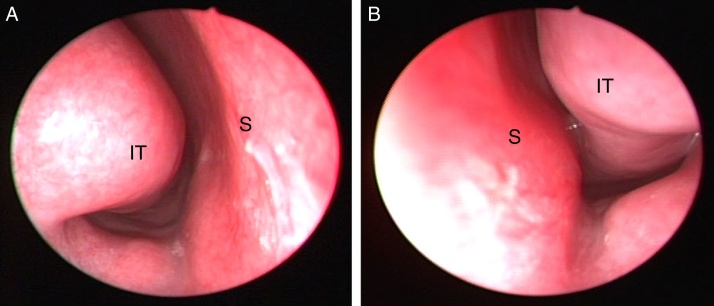
Preoperative nasal endoscopic findings show enlargement of the left inferior turbinate. (A) Right side and (B) left side of the nasal cavity (S, nasal septum; IT, inferior turbinate).

**Figure 2:**
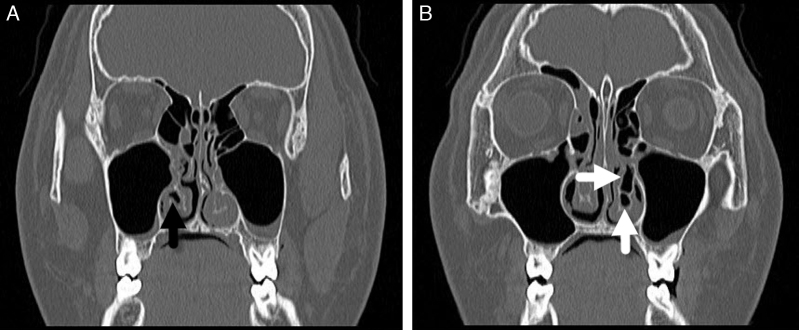
Computed tomography (CT) images of the patient. (A, B) Coronal view of CT images shows pneumatization of both the inferior turbinate and hypertrophy of the left inferior turbinate (black arrow, right inferior concha bullosa (ICB); white arrows, left ICB).

**Figure 3:**
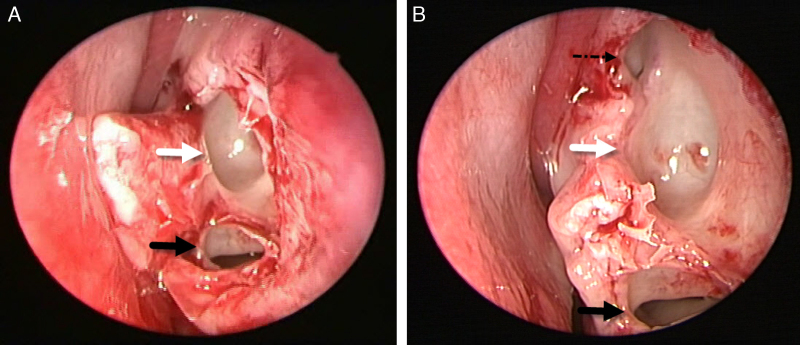
Intraoperative findings. Figures show upper ICB connecting with the natural ostium of the maxillary sinus and lower ICB (left) (black arrow, lower ICB; white arrow, upper ICB; dotted arrow, natural ostium of maxillary sinus).

A 14 year-old male presented to this otolaryngology department with nasal obstruction, chronic headache, and purulent nasal discharge, which had persisted for several years. He denied any history of nasal allergy, trauma, or sinus surgery. Rigid nasal endoscopy showed enlargement of left inferior turbinate (Fig. 1A and B). For further examination, non-contrast computed tomography (CT) of the osteomeatal unit was performed. CT revealed bilateral pneumatization of the inferior turbinate and hypertrophy of the left inferior turbinate (Fig. 2A and B). Two concha bullosa were located at the posterosuperior and posteroinferior portions of the left inferior turbinate (Fig. 3A). The upper ICB was connected to the natural ostium of the maxillary sinus (Fig. 3B). The patient underwent endoscopic surgery including left middle meatal antrostomy, left inferior turbinectomy, and lateral out-fracture of both inferior turbinates under general anesthesia. At the follow-up, six months post-surgery, the patient's symptoms had significantly improved and he was free of nasal complaints.

## Discussion

The inferior turbinate is a separate bone originating from the lateral nasal wall. It is the largest turbinate and is covered with a thick mucous membrane that contains a cavernous plexus.^4^ Mainly, it contributes to thermoregulation, humidification, and filtration of inspired air. Embryologically, the inferior turbinate develops from the prechordal plate. At week six of fetal development, a series of elevations appear on the lateral wall of the nose that will ultimately form the turbinates, including the inferior turbinate.^5^ The permanent inferior turbinate originates from maxilloturbinal formations, and is a completely separate structure located at the inferior part of the ethmoid ridges.^4^ ICB is a rare anatomical variant of the osteomeatal complex. In the literature ‘CT appearance of pneumatized inferior turbinate’, only 60 (0.03%) out of 59,238 patients were found to have pneumatized inferior turbinate. Among them, pneumatized inferior turbinate was unilateral in 14 (88%) patients and bilateral in two (12%) patients. Another literature ‘Pneumatization of the Inferior Turbinate: Incidence and Radiologic Appearance’ shows pneumatized inferior turbinate was found in one out of 250 cases (0.4%): seven of these were unilateral and three were bilateral.^2^, ^3^ Several hypotheses have been suggested regarding the mechanism(s) of ICB formation. The first hypothesis suggests that ICB arises from the ossification of the chondral framework of the inferior concha to a double lamella during fetal life, with the misinvagination of the epithelium into the double lamella causing the formation of concha bullosa.^6^ The second hypothesis suggests that air-filled cavities are intimately associated with the inferior turbinate attachment, which is caused by maxillary sinus disease.^7^ The third hypothesis suggests that in fetal life, maxillary sinus pneumatization extends into the inferior turbinate, and this finding can be readily seen in axial CT.^2^ For the majority of ICB cases, connectivity with the maxillary sinus is observed.^4^ In general, ICB is asymptomatic and diagnosis is usually incidentally detected with CT. However, if the ICB is large, it may obstruct the nasal cavity and consequently result in headaches and sinusitis. The severity of the symptoms caused by ICB is closely related to the extent of pneumatization. Nasal obstruction or congestion is also associated with inferior turbinate hypertrophy. Therefore, it is important to differentiate between inferior turbinate hypertrophy and ICB, as the latter can easily be misdiagnosed as the former. Although the use of vasoconstrictor drugs may be helpful in distinguishing the two, a definitive diagnosis is only achieved by CT of the paranasal sinus. Treatment is not necessary for cases of asymptomatic ICB and should only be implemented when ICB is symptomatic. The goals of treatment are to maximize the nasal airway and to minimize the symptoms and complications. Nasal symptoms and complications may respond to medical therapy, such as vasoconstrictor drugs or steroid nasal spray, but typically respond best to surgery. The surgical techniques used include out-fracture of the inferior turbinate, crushing of the ICB with concha bullosa crusher, excision of the free edge of the inferior turbinate with angled scissors, submucosal turbinoplasty with radiofrequency coblator, and resection of the ICB lateral lamella.^1^ However, if there is a connection between CB and the maxillary sinus, lateral resection of the turbinate may cause inferior meatal antrostomy, which could ultimately result in recirculation problems.^8^ Furthermore, if turbinitis is accompanied by ICB, the infection should be treated before surgery. With the widespread use of imaging instruments such as CT, surgeons are able to get detailed information about nasal cavity and paranasal sinuses before surgery and to anticipate points of safe entry into the lumen of concha bullosa.^9^

## Conclusion

Pneumatization of the inferior turbinate, referred to as ICB, is a rare intranasal anatomic variation of the lateral nasal wall. It is usually asymptomatic and diagnosed incidentally by CT; occasionally, it results in nasal obstruction, recurrent sinusitis, and headaches. In this study, in addition to a review of the literature, a case of bilateral pneumatization of the inferior turbinate (ICB connected with the natural ostium of the maxillary sinus) was presented, which resulted in nasal obstruction and need for surgical treatment.

## Conflicts of interest

The authors declare no conflicts of interest.
